# You don't know what you don't know; using high school outreach to improve awareness of bioscience-based careers and higher education

**DOI:** 10.1016/j.crphys.2025.100151

**Published:** 2025-06-11

**Authors:** Christine Greensmith, David Greensmith

**Affiliations:** a De La Salle School, Saint Helens, WA10 4QH, United Kingdom; b Biomedical Research and Innovation Centre, The University of Salford, Salford, M5 4WT, United Kingdom

**Keywords:** Outreach, STEM, Higher education, Social disadvantage, Widening participation

## Abstract

When considering the diversity of students in higher education, an important but frequently overlooked characteristic is socioeconomic background. It is well known that those from socially disadvantaged backgrounds are less likely to progress to higher education, and accounts for an associated lack of diversity in the STEM workplace. The reasons for this are many and complex, though a lack of awareness of STEM-based education thence career pathways among secondary school learners remains a key contributor. To address this, we designed an adaptable and scalable high school outreach programme that sought to raise STEM (with a focus on bioscience) awareness through events that could be readily adapted to meet high school needs and resource constraints. Learner perceptions were recorded using a pre- and post-event questionnaire. Following the event, awareness of bioscience-based careers and the required prerequisite skills and qualifications were increased by 58 and 53 % respectively. The degree to which learners were considering a bioscience-based career was increased by 43 %. Though interest in attending university to study STEM was unaltered, awareness of the qualifications required to progress to university was increased by 58 %. These findings suggest that outreach events are an effective way to raise general awareness of STEM-based higher education learning thence careers and highlight the importance of tailoring outreach events to meet school and learner needs.

## Highlights

• We designed a high school outreach event to raise awareness of STEM careers and HE.
• Our outreach events increased interest in bioscience-based careers.
• Learner understanding of higher education was improved.
• Curriculum-aligned learning was enhanced.

## 1 Introduction

At The University of Salford, we are proud of our diverse student population. For instance, in the academic year 2020–21, 57 % of Salford students were female, 30 % mature, 30 % of BAME origin and 26 % declared a disability, (Equality and Diversity, 2022). For STEM-based subjects specifically, in the same period, while 45 % of students were of BAME origin, fewer students were female (32 %) and fewer declared a disability (20 %), a pattern that is reflected by similar lack of diversity in the STEM-workplace (Woolston, 2021; Inquiry into equity in the STEM workforce, 2021). When considering diversity, one characteristic that is frequently overlooked is socioeconomic background. This is an important oversight. To take The University of Salford as an example, 57 % of students are from Greater Manchester which has one of the highest concentrations of deprived localities in the United Kingdom (Noble et al., 2019; Nokes, 2019). Of course, this pattern is likely true of any university with high intakes from deprived areas (Britton et al., 2021). Despite this, many organisations do not collect data on the socio-economic background of staff. Yet, where this data exists, it reflects an associated and similar lack of diversity in the workplace (Friedman et al., 2015; The Bridge Group, 2022; Social Mobility Commission, 2019).

In simple terms, if a secondary school learner is to enter a STEM-based career, they must (1) be aware those careers exist and (2) understand the importance of prerequisite further then higher education pathways so they can make appropriate learning choices. However, socially disadvantaged learners often lack this insight meaning individuals who *might* excel in STEM and aspire to enter associate careers do not do so simply because they are unaware these pathways exist. You don't know what you don't know.

Evidence for this comes from progression to higher education statistics. It is well known that those from socially disadvantaged backgrounds are less likely to progress to higher education and even less likely to progress to high tariff institutions (Bolton et al., 2023; EngineeringUK, 2022). The reasons for this are many (Jury et al., 2017; Forsyth et al., 2003; Hoskins et al., 2017). Some examples of relevance here include that socially disadvantaged learners are often first in their family to (potentially) attend university and they are less likely to have family or community connections to STEM or those with knowledge of STEM-based careers. Furthermore, they are less likely to engage with informal learning (for example, visits to science museums), benefit from outreach or have access to robust career support (Campaign for Science and Engineering, 2021; Campaign for Science and Engineering, 2022; Bolton and Lewis, 2024). These issues compound what is often a general lack of awareness of STEM-career scope among teachers and mentors (Knowles et al., 2018).

To begin to address these issues, many universities engage in outreach by running events in local secondary schools (Johnson et al., 2019; Heaslip et al., 2020). While positive, frequently, these events are complex or have a rigid format. However, constraints such as university staff availability and resource limitations in secondary schools makes delivery of a standardised outreach event problematic. If we are to engage as many learners as possible in a given outreach programme, the associated events must be (1) relatively simple; for ready and responsive implementation and (2) flexible; so they meet the needs and constraints of all involved.

Furthermore, while most events are well meaning and primarily designed to improve widening participation (Johnson et al., 2019; Heaslip et al., 2020) and attitudes towards STEM (Vennix et al., 2018), many also serve some need of the university, for instance recruitment (Marr, 2013). Many may inadvertently hard-sell (Holmwood, 2016) or give the impression that higher education learning pathways are superior, which we feel is the wrong message. In doing this, the needs of the secondary school and what *they* would like gain from outreach (for example, reinforced learning or exposure to advanced learning materials and technologies (Panizzon et al., 2018)) may be assumed or not considered.

To address this, we designed a modular, flexible outreach event format that sought to (1) raise awareness of bioscience-based careers and university-based education pathways while (2) serving the secondary schools by aligning activities to specific needs. The objective was not to disparage other career options or *persuade* learners to make choices that lead to university. Rather, to raise the awareness required to make informed decisions and ensure that learners did not miss the opportunity to progress to university thence associated careers simply because they did not know those pathways existed.

## 2 Methods

### 2.1 Development of the Salford Schools Network

To establish a more tangible and established relationship with the schools involved in the programme, we developed the Salford Schools Network. This included several local (Salford area and localities with high indices of social deprivation (Noble et al., 2019; Nokes, 2019)) secondary schools and placed us in direct contact with teaching staff. The network also provided convenient communication channels to facilitate awareness of and dissemination of outcomes from the programme. This permitted organic expansion of the programme's scale and reach.

### 2.2 Event format

The programme included a series of events at schools in the Salford school network. The precise scale and format of each event varied according to school needs and constraints but generally included a circuit of activity stations in arbitrary order. Each activity lasted for 20 min and were suitable for around 10 students. As such, the cohort was split into groups that progressed through each station in turn such that by the end of the event all students engaged with all activities. The authors are pleased to provide full details on event format and associated resources by request.

### 2.3 Event planning

To design a given event at a given school required 3 fundamental considerations; (1) the school's need (number and level of students, learning outcomes), (2) school constraints (time available, availability of wet/dry labs and utilities) and (3) university constraints (staff and resource availability). Given this, we developed a modular approach.

Here, we defined 15 learning themes, for example microscopy, physiology, anatomy, microbiology, haematology, health and disease, careers, etc. Each theme contained several discrete activities designed by staff. For instance, activities within the anatomy theme included various dissections or interaction with models. Those within the physiology theme included use of physiology teaching kits to measure ECG, ventilation or neurological state, etc. (see Table S1 in supplementary material for a full list). Collectively this produced a large pool of activities from which we could fluidly draw for a given event.

Before each event, we asked the school to complete a form. This captured basic information such as the number of participants, time available, risk assessment status and permissions. Furthermore, it ascertained to what extent specialist spaces such as wet labs, and utilities such as water, waste and power would be available. The form also asked the school to select the learning *themes* they would like the event to include.

This information was then integrated with university staff availability and activities selected from the pool accordingly. When combined, those activities formed an event that met the schools learning needs and constraints yet that was convenient to deliver.

### 2.4 Event coordination

On the day, university staff arrived at the school to set up the activity stations. Each was run by an expert in the field aligned to the activity. Undergraduate volunteers were also recruited to engage with learners, assist with activities and to ensure smooth running. We provided each volunteer with dedicated training on practical and professional expectations, forming a pool from which we could proportionally draw according to the size of the event.

### 2.5 Data collection and analysis

Following approval from The University of Salford Ethics Panel, learners were asked to complete a pre-event questionnaire. This presented five statements.

1. I know what bioscience-based careers are
2. I know what skills and qualifications are required to enter a bioscience-based career
3. I am considering a bioscience-based career
4. I am considering studying science at university
5. I know what qualifications are required to go to university.

Learners were asked to respond with strongly agree, agree, disagree or strongly disagree then asked to complete the same questionnaire post-event.

To quantify thence statistically analyse shifts in perception, each qualitative response option was assigned a value: strongly agree; 4, agree; 3, disagree; 2, strongly disagree; 1. Perceptions are presented as mean ± SEM score. Statistical significance was examined using *t*-test and accepted when p < 0.05. Data combines responses from two events at separate schools.

## 3 Results

### 3.1 The qualitative impact of outreach on secondary school learner perceptions of bioscience-based careers

**Fig. 1:**
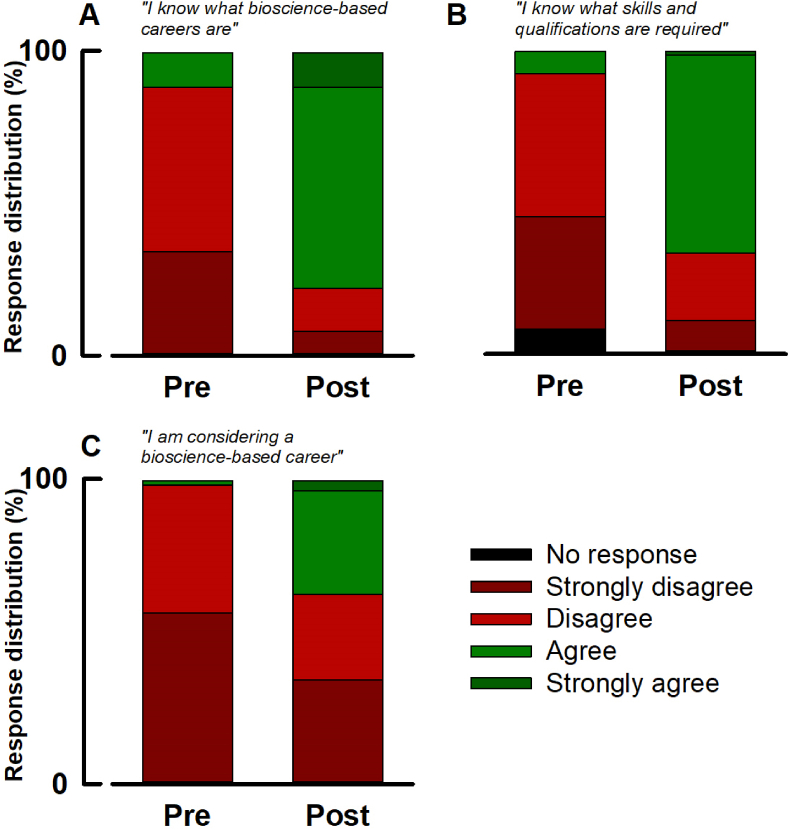
**The impact of outreach on secondary school learner perceptions of bioscience-based careers.** All figures show the distribution of responses to questions designed to probe awareness of bioscience-based careers pre and post the event. For all, learners were asked to rank their perception according to the scale represented by the legend. **A;***“I know what bioscience-based careers are”***B;***“I know what skills and qualifications are required to enter a bioscience-based career”***C;***“I am considering a bioscience-based career”*.

To qualitatively assess the impact of the outreach event on perceptions, learners were asked to complete a pre-event questionnaire, then again post-event. The first three questions (Fig. 1) were designed to probe the learners’ general perceptions of bioscience-based careers. Before the event only 12 % of responders agreed (no strong agrees) with the statement “*I know what bioscience-based careers are”*. This increased to agree; 66 % and strongly agree; 12 % post event. To the statement *“I know what skills and qualifications are required to enter a bioscience-based career”*, only 8 % of leaners responded in agreement before the event. This increased to agree; 65 % and strongly agree; 2 % post event. Before the event only 2 % of responders agreed with the statement *“I am considering a bioscience-based career”* which increased to agree; 34 % and strongly agree; 4 % post event.

### 3.2 The qualitative impact of outreach on secondary school learner perceptions of university

**Fig. 2:**
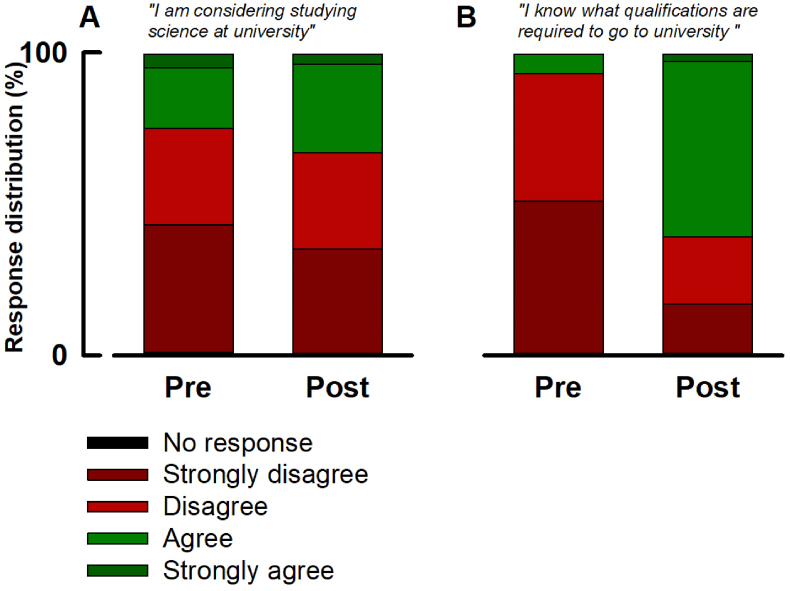
**The impact of outreach on secondary school learner perceptions of university.** Both figures show the distribution of responses to questions designed to probe awareness of university pre and post the event. For all, learners were asked to rank their perception according to the scale represented by the legend. **A;***“I am considering studying science at university”***B;***“I know what qualifications are required to go to university”*.

The remaining questions (Fig. 2) were designed to probe the learners’ perceptions of university. Before the event only 20 % of responders agreed and 5 % strongly agreed with the statement “*I am considering studying science at university”*. This distribution shifted to agree; 29 % and strongly agree; 4 % post event. Before the event only 7 % of responders agreed (no strong agrees) with the statement “*I know what qualifications are required to go to University”*. This increased to agree; 58 % and strongly agree; 3 % post event.

### 3.3 Quantitative analysis of the impact of outreach on secondary school learner perceptions

**Fig. 3:**
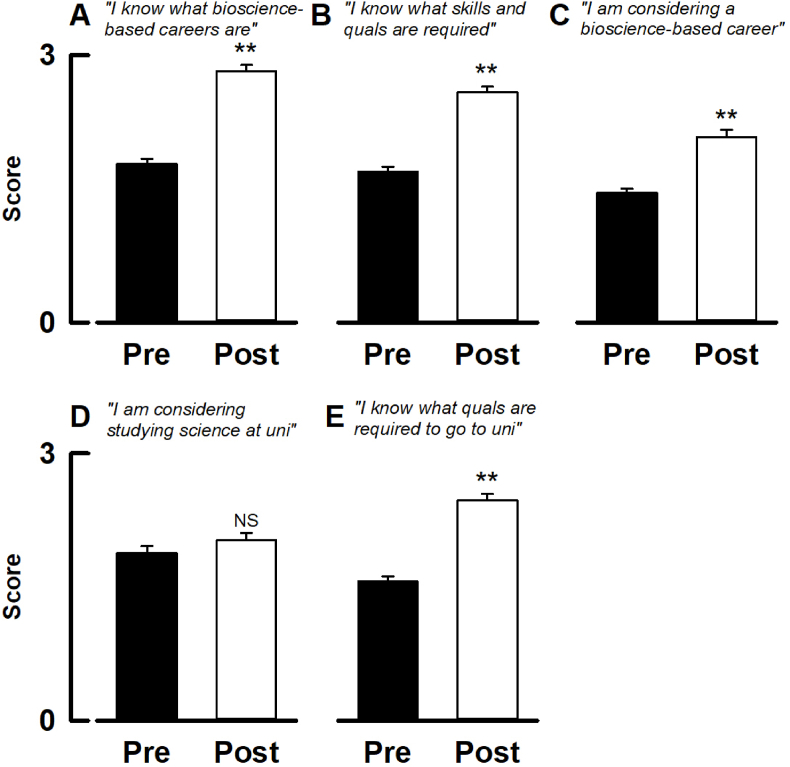
**Quantitative analysis of the impact of outreach on secondary school learner perceptions.** Bars represent mean ± SEM response score pre and post the event. n = 123 responses. **A;***“I know what bioscience-based careers are”***B;***“I know what skills and qualifications are required to enter a bioscience-based career”***C;***“I am considering a bioscience-based career”***D;***“I am considering studying science at university”***E;***“I know what qualifications are required to go to university”*. ∗∗: p < 0.001. NS: no statistical significance.

To permit quantitative comparison of pre- and post-event perceptions presented in Fig. 1, Fig. 2, responses were allocated a score then subjected to statistical analysis, the outputs of which are presented in Fig. 3. Following the event, the score associated with the statement “*I know what bioscience-based careers are”* was increased by 58 % (pre; 1.78 ± 0.06, post; 2.82 ± 0.07, n = 123, p < 0.001) while that associated with “I know what skills and qualifications are required to enter a bioscience-based career” was increased by 53 % (pre; 1.69 ± 0.06, post; 2.58 ± 0.06, n = 122, p < 0.001). The score associated with the statement *“I am considering a bioscience-based career”* was increased by 43 % (pre; 1.46 ± 0.05, post; 2.08 ± 0.08, n = 123, p < 0.001). The event had no significant effect on the score associated with the statement *“I am considering studying science at university*” (pre; 1.88 ± 0.08, post; 2.02 ± 0.08, n = 123, p = 0.17) though did increase the score associated with the statement *“I know what qualification are required to go to university*” by 58 % (pre; 1.56 ± 0.06, post; 2.47 ± 0.07, n = 123, p < 0.001).

## 4 Discussion

### 4.1 What were the learners’ perceptions of bioscience-based careers and university before the event?

The pre-event questionnaire sought to elucidate baseline perceptions. The questions represented by Fig. 1 were designed to probe the learners’ general awareness of bioscience-based careers. Before the event, 88 % of learners disagreed to an extent with the statement “*I know what bioscience-based careers are”* (Fig. 1A) thus demonstrating a fundamental lack of awareness. It is therefore little surprise that 84 % of learners were unaware of the skills and qualifications required to enter a bioscience based-career and that only 2 % saw a bioscience-based career as *an* option (Fig. 1B and C respectively). While we did not probe interest in the subject area *per se*, the association between general STEM unawareness and disinterest in associated careers is well known, and perhaps unsurprising (Kudenko et al., 2016; Spyropoulou et al., 2020). What these data *do* tell us, is that baseline awareness was low in this cohort.

Nonetheless, it may have been that this unawareness was contained to biosciences, so the questions represented by Fig. 2 were designed to probe the learners’ awareness of university-based STEM pathways more generally. Indeed, 25 % of respondents agreed with the statement *“I am considering studying science at university”* (Fig. 2A). One might argue this is encouraging and given the relatively early stage of the learner, might underestimate future progression. Even so, if used as an *indicator* of those who *might* progress to university, this proportion remains low by national standards. For instance, on average 32 % of learners from the lowest POLAR group (Q1) progress to University as do 30 % of those eligible for free school meals (Bolton et al., 2023). Whether this disinterest is entirely due to unawareness, or, to some extent that learners have already committed to other career aspirations (Smith et al, 2010) or are apprehensive (Lowe and ACook, 2003) remains to be elucidated. In any case, that only 7 % of respondents understood the prerequisite learning requirements for progression to higher education suggests a general lack of awareness is a key contributor.

These findings reinforce the idea that many learners may not progress to university thence related careers simply because (1) they are unaware such pathways exist and/or (2) the requirements are not clear. That “they don't know what they don't know”, in many cases may lead to poorly informed further then higher education and career choices. Of course, we are *not* suggesting that all students ought to go to university or that non-university-based education and careers are inferior. We are simply saying that any choice ought to be adequately informed, and for that, learners must be fully aware of options and associated prerequisites.

### 4.2 Were the learners’ perceptions of bioscience-based careers and university shifted by the event?

Following the event, the learners were asked to complete the same questionnaire. Fig. 1, Fig. 2 qualitatively indicate positive shifts in perception associated with each statement. To permit quantitative comparison of pre- and post-event awareness, responses were allocated a score then subjected to statistical analysis (Fig. 3).

Fig. 3A and B demonstrate that the events increased awareness of bioscience-based careers and associated prerequisite skills and qualifications by 58 and 53 % respectively. Importantly, this produced a 43 % increase in bioscience-based career interest (Fig. 3C). Interestingly, the events did not *significantly* increase the *immediate* interest in STEM at university (Fig. 3D), but did produce a considerable (58 %, Fig. 3E) increase in awareness of university prerequisites. Given this, it may be that we simply honed – towards biosciences – the aspirations of learners *already considering* an academic learning then career path. In any case, general awareness of bioscience-based careers and university-based pathways *was* increased, which was the objective of the exercise. It is the ability to make *informed* career choices that is key, whether those choices lead to university and STEM-based careers or not (Cantrell et al., 2009).

### 4.3 Might this make a difference to learners?

The positive effects of outreach on widening participation and STEM awareness are well documented (Johnson et al., 2019; Heaslip et al., 2020; Vennix et al., 2018). Our outreach events hoped to raise awareness among learners in areas with high indices of social disadvantage (Noble et al., 2019; Nokes, 2019) - thus improve associated widening participation - in ways that were also useful to the schools.

As discussed in 4.2, our findings suggest that awareness of bioscience-based careers and the pre-requisites required to enter higher education *were* increased. However, given we saw no *significant* increase in the level of interest in STEM at university, one might argue that here, awareness will not translate to progression to higher education thence associated careers thus make a tangible difference to widening participation.

However, we ought to consider two things. (1) though not statistically significant, interest in university did increase by 8 %. We must remember that the questionnaire reflects the perception of individuals, so this tells us that several learners who were not before, are now *considering* university. To those individuals, the impact is very real. (2) The objective of these events was *not* to drive students to university. Rather, to ensure they were simply aware of the option.

Here, we argue that the second point is most important. Secondary school pupils are still at a relatively early stage, and we know that for many, aspirations change as they and their learning develop (Aschbacher et al., 2010; DesJardins et al., 2019; Tang et al., 2008). To a large extent, the breadth of scope of those aspirations is dependent on insight, which in socially disadvantaged learners may be limited by a lack of family and community connections to STEM and STEM careers, informal learning and access to robust career support (Campaign for Science and Engineering, 2021; Campaign for Science and Engineering, 2022 Bolton and Lewis, 2024; Knowles et al., 2018); you don't know what you don't know. Our data suggests that the events *did* provide insight through raised awareness which ought to expand the scope of potential aspirations in the learners.

Of course, this simply adds a STEM-based career *option* to many others under consideration by the learner. Even with this information, it remains the case that learners may or may not develop an interest in university as they progress through their remining time at school then college. In any case we can be more confident that any choices made are better informed. Crucially, we reduce instances where a learner misses the opportunity to enter a STEM-based career that may be ideally suited to them simply because they do not know it exists. Or, where options are limited by further education choices that prevent or delay access to higher education should that desire then develop.

### 4.4 Wider advantages

When compared to relatively advantaged counterparts, many socially disadvantaged learners who develop an interest in higher education must overcome more barriers to progress to university despite being as qualified (Bolton et al., 2023; EngineeringUK, 2022; Jury et al., 2017; Forsyth et al., 2003; Hoskins et al., 2017). Once at university, they then face another challenge in that they may be behind the graduate capital curve, limiting their prospects. Therefore, initiatives that seek to enhance graduate capital are important, especially for cohorts that contain high proportions of students from socially disadvantaged backgrounds (Namvar et al., 2023). While there are many ways to do this (Namvar et al., 2023; Namvar et al., 2021; Namvar et al., 2021), engaging with outreach events provides a particularly effective way for undergraduate students to develop a range of skills (Smith et al., 2010). For our own volunteers, we further developed the opportunity by providing a dedicated training programme which sought to enhance delivery, communication and professional skills.

While in principle, any well-designed outreach event ought to raise intended awareness in learners; our objective, we argue that the needs of the school are equally important. While the activities that formed our events were an ideal vehicle to raise awareness, they were also designed for, then selected by the schools to provide additional and enhanced learning that was highly relevant to school curricula. In this respect, anecdotal feedback was favourable and accounted for programme's rapid expansion.

## 5 Summary

When it comes to making learning and career choices, high school pupils frequently don't know what they don't know. Our outreach events increased awareness of and interest in bioscience-based careers, and improved understanding of higher education in ways that were also useful to the secondary school. We hope this will better inform the career choices that learners make, whatever those choices may be.

## CRediT authorship contribution statement

**Christine Greensmith:** contributed to the acquisition and analysis of data and preparation of the manuscript. **David Greensmith:** conceived and led the programme of work, contributed to the acquisition and analysis of data and preparation of the manuscript.

## Declaration of competing interest

The authors declare that they have no known competing financial interests or personal relationships that could have appeared to influence the work reported in this paper.

## Data Availability

Data will be made available on request.
