# Supplementary material for: You don't know what you don't know; using high school outreach to improve awareness of bioscience-based careers and higher education
Source: Curr Res Physiol. 2025 Jun 11;8:100151. doi: 10.1016/j.crphys.2025.100151 (PMC12221277; doi:10.1016/j.crphys.2025.100151)
Supplement: Multimedia component 1 [file mmc1.docx]

**Supplementary material**

Table S1 expands on the learning themes and associated example activities briefly presented in section 2.3.

| **Table S1: Learning themes and example activities** | |
| --- | --- |
| **Theme** | **Example activities** |
| Microscopy | Basic microscopy theory and microscope use.  Specimen and slide preparation.  Sample analysis.  Histological drawings. |
| Physiology (how the body works) | Measurement of heart / lung / brain activity using ADI teaching kits.  Thermography.  Capillaroscopy. |
| Pathophysiology (disease) | Neurobiology and dementia workshop.  Cardiovascular disease workshop.  Muscle disease & arthritis workshop.  Cancer workshop. |
| DNA | DNA extraction and / or fingerprinting |
| Haematology | Blood cell count and diagnostics. |
| Anatomy / dissections | Anatomy model workshops.  Dissections (heart / lung / brain / etc). |
| Microbiology | Plate preparation and microscopy  Self-swabs and plating (incubation and results reported subsequently) |
| Evolutionary biology | Comparative specimen / fossil workshops.  Fossil 3D scanning. |
| Chemistry | Nano particle workshop. |
| Ecology | Aquatic ecosystems workshop.  Marine biology workshop.  Biodiversity workshop. |
| Digital health care | Smartphones and health information workshop. |
| STEM-based crafting | Modelling (anatomy).  Cake decorating (anatomy).  Hat production (brain). |
| Flash talks | Topical and inspiring STEM-based talks by undergraduate students. |
| Careers in research | Workshops designed to raise awareness of research-based careers and associated prerequisites. |
| Clinical careers | Workshops designed to raise awareness of medicine, dentistry, etc and associated prerequisites. |
